# Transcript levels of different cytokines and chemokines correlate with clinical and endoscopic activity in ulcerative colitis

**DOI:** 10.1186/1471-230X-9-13

**Published:** 2009-02-09

**Authors:** Alexandra Zahn, Thomas Giese, Max Karner, Annika Braun, Ulf Hinz, Wolfgang Stremmel, Robert Ehehalt

**Affiliations:** 1 Department of Gastroenterology, University Hospital Heidelberg, Im Neuenheimer Feld 410, 69120 Heidelberg, Germany; 2 Institute of Immunology, University Heidelberg, Im Neuenheimer Feld 305, 69120 Heidelberg, Germany; 3 Unit for Documentation and Statistics of the Department of Surgery, University of Heidelberg, Heidelberg, Germany

## Abstract

**Background:**

A definition of disease activity in ulcerative colitis (UC) is difficult. The clinical activity index (CAI) is only an indirect assessment tool of bowel inflammation and the endoscopic activity index (EAI) sometimes cannot reflect the severity of disease to the full extent. Therefore, there is a need for an objective means to quantify inflammatory activity in mucosal biopsies. In our study, we wanted to examine the correlation between transcript levels of interleukin 8 (CXCL8), interferon γ inducible protein 10 (CXCL10), myeloid-related protein 14 (calgranulin B), macrophage inflammatory protein 2 α (CXCL2) with CAI and EAI in UC.

**Methods:**

Cytokine and chemokine transcripts were quantified using real-time PCR in 49 mucosal biopsies from 27 different patients with UC. Cytokine transcript levels were correlated with CAI and EAI.

**Results:**

There was a statistically significant positive correlation between CXCL8 (r = 0.30; p < 0.05), CXCL10 (r = 0.40; p < 0.02), calgranulin B (r = 0.36; p < 0.03), CXCL2 (r = 0.31; p < 0.05) and CAI. Concerning EAI significant positive correlations for CXCL8 (r = 0.37; p < 0.02), CXCL10 (r = 0.33; p < 0.04), calgranulin B (r = 0.31; p < 0.05) and CXCL2 (r = 0.44; p < 0.05) were found. Low clinical and endoscopic activity was accompanied by low cytokine levels whereas high CAI and EAI were associated with high cytokine levels.

**Conclusion:**

From our data, we conclude that real-time PCR quantification of CXCL8, CXCL10, calgranulin B and CXCL2 in colonic biopsies is a simple and objective method for grading inflammation of intestinal mucosa in UC. CXCL8, CXCL10, calgranulin B and CXCL2 might be used as biomarkers and thus as an objective tool especially in clinical trials to evaluate anti-inflammatory and immunomodulatory regimens.

## Background

Inflammatory bowel disease (IBD) like ulcerative colitis (UC) and Crohn's disease (CD) are characterized by a relapsing and remitting clinical course. Disease activity and severity are variable and include both segmental processes with slight impairment of state of health and pancolitis with extensive gastrointestinal and systemic symptoms. Thus, the definition of disease activity in UC is often difficult. The clinical activity index (CAI) is only an indirect assessment tool of bowel inflammation and the endoscopic activity index (EAI) is sometimes unable to reflect the severity of disease to the full extent.

Cytokine and chemokine mRNA expression profiles in UC have been characterized in former studies [1-3] and interleukin 8 (CXCL8), interferon γ inducible protein 10 (CXCL10), myeloid-related protein 14 (calgranulin B) and macrophage inflammatory protein 2 α (CXCL2) were identified as important inflammatory mediators. However, transcript levels of these cytokines and chemokines in the blood can not reflect the inflammatory activity in the gut accurately [2,3]. But a correlation between mucosal mRNA levels of specific cytokines and chemokines measured by real-time PCR and mucosal inflammation has been shown for CD patients and in patients with ileopouch anal anastomosis (IPAA) [4,5]. However, so far no data concerning the correlation between transcript levels of the above mentioned selected pro-inflammatory cytokines CXCL8, CXCL10, calgranulin B and CXCL2 in mucosal biopsies and disease activity indices of UC patients exist. Previous studies either evaluated changes in other than the above mentioned cytokines/chemokines like Raddatz et al. [6] who showed a positive correlation between interleukin 6 (IL-6) mRNA transcript levels and CAI in patients with pancolitis or used methods like Northern Blot analysis [7], in situ hybridization [8], ELISA [7,9-12] or immunohistochemistry [8] which are not considered reliable for quantification of mucosal cytokine gene expression. As an adequate treatment of UC requires an objective assessment of mucosal inflammation, there is a need for a simple and objective means to quantify inflammatory activity. The recent development of real time PCR methodology should allow a routine and reliable quantification of PCR products. Therefore, we measured the transcript levels of CXCL8, CXCL10, calgranulin B and CXCL2 in biopsies of UC patients in this study and correlated them to CAI and EAI.

Thus, the aim of this study was to establish several biomarkers which can reflect mucosal inflammation in UC.

## Methods

### Patients

All biopsies were taken from patients who participated in one of our clinical studies [13,14]. The biopsies were taken from the colon section 30–40 cm ab ano to get a standardization for our study. Our studies were approved by the institutional Ethics Committee of the University of Heidelberg. CAI and EAI were assessed according to Rachmilewitz [15] (tables 1 and 2). Altogether, 49 biopsies from 27 different patients were examined. In table 3 patients' sociodemographic and clinical data are given. Nineteen patients were male, eight female. Patients' mean age was 37 years (range, 18–53 years). Patients' disease duration averaged out at 9 years (range, 1–23 years). Seventeen of our patients had a pancolitis, nine a left sided colitis and one patient had only proctitis. In table 4 the CAI (mean CAI, 6; range, 0–14) and the EAI (mean EAI, 7; range 2–10) assigned to each biopsy at the time of sampling are shown. Three patients had extraintestinal manifestations of UC, namely arthritis. 24 patients received drug treatment. A single treatment regimen with prednisolone was given to 7 patients, a single treatment regimen with 5-aminosalicylic acid (5-ASA) to 5 patients. 6 patients received prednisolone and 5-ASA, 3 patients received prednisolone and azathioprine and 3 patients were treated with prednisolone, 5-ASA and azathioprine. If one patient was included in our study twice, biopsies were taken at two different time points at intervals of at least 3 months.

**Table 1:** Clinical Activity Index (CAI) according to Rachmilewitz.

Number of stools weekly	
< 18	0
18–35	1
36–60	2
> 60	3

**Blood in stools (based on weekly average**)	

None	0
Little	1
A lot	2

**Investigator's global assessment of symptomatic state**	

Good	0
Average	1
Poor	2
Very poor	3

**Abdominal pain/cramps**	

None	0
Mild	1
Moderate	2
Severe	3

**Temperature due to colitis**	

< 38°C	0
> 38°C	1

**Extraintestinal manifestations**	

Iritis	3
Erythema nodosum	3
Arthritis	3

**Laboratory Findings**	

Sedimentation rate > 50 mm in 1st hour	1
Sedimentation rate > 100 mm in 1st hour	2
Haemoglobin < 100 g/L	4

**Table 2:** Endoscopic Activity Index (EAI) according to Rachmilewitz.

Granulation scattering reflected light	
No	0
Yes	2

**Vascular pattern**	

Normal	0
Faded/disturbed	1
Completely absent	2

**Vulnerability of mucosa**	

None	0
Slightly increased (contact bleeding)	2
Greatly increased (spontaneous bleeding)	4

**Mucosal damage (mucus, fibrin, exudate, erosions, ulcer)**	

None	0
Slight	2
Pronounced	4

**Table 3:** Patients' characteristics

Number of Patients	27
Sex (m/f)	19/8
Age (years)	37 (18–53)
Disease Duration (years)	9 (1–23)
Disease Localisation: Pancolitis/Left sided colitis/Proctitis	17/9/1
Extraintestinal Manifestations (arthritis)	3

Values are given as absolute numbers and means. The values in brackets give the extreme values.

**Table 4:** Biopsies' characteristics

Number of Biopsies	49
Mean CAI	6 (0–14)
Mean EAI	7 (2–10)

Values are given as absolute numbers and means. The values in brackets give the extreme values.

### Real-Time RT-PCR

Biopsies were collected in RNAlater (Ambion, Austin, TX, USA), and stored at -20°C until analysis. Tissue was disrupted by one run with the RiboLyser (ThermoHYBAID, Heidelberg) in lysing matrix „D“ tubes (Q-BIOgen, Heidelberg) containing 400 μl lysis buffer from the MagnaPure mRNA Isolation Kit II (ROCHE Diagnostics, Mannheim). The RiboLyser tubes were centrifuged at 4°C for 1 min at 13000 rpm. 300 μl of the lysate was collected and mixed with 600 μl capture buffer containing oligo-dT. After centrifugation at 13000 rpm for 5 min, 880 μl of this mix was transferred into a MagnaPure sample cartridge and mRNA was isolated with the MagnaPure-LC device using the mRNA-II standard protocol. The elution volume was set to 50 μl.

An aliquot of 8.2 μl mRNA was reversely transcribed using AMV-RT and oligo-(dT) as primer (First Strand cDNA synthesis kit, Roche) according to the manufacturers instructions in a thermocycler. After termination of the cDNA synthesis, the reaction mix was diluted to a final volume of 500 μl and stored at -20°C until PCR analysis.

Primer sets optimized for the LightCycler (RAS, Mannheim Germany) were developed and purchased from SEARCH-LC GmbH, Heidelberg. The PCR was performed with the LightCycler FastStart DNA Sybr GreenI kit (RAS) according to the instructions provided in the parameter specific kits. To control for specificity of the amplification products, a melting curve analysis was performed. No amplification of unspecific products was observed. The copy number was calculated from a standard curve, obtained by plotting known input concentrations of four different plasmids at log dilutions to the PCR-cycle number (CP) at which the detected fluorescence intensity reaches a fixed value. This approach dramatically reduced variations due to handling errors over several logarithmic dilution steps.

To correct for differences in the content of total RNA, the calculated copy numbers were normalized according to the average expression of two housekeeping genes, β-Actin and Cyclophilin B. Values were thus given as input adjusted copy number per μl of cDNA.

### Statistical analysis

Statistical analysis was performed using SAS software (Release 9.1, SAS Institute, Cary; NC, USA). Results are described as medians, means and ranges. Correlations between cytokine/chemokine transcript levels and CAI and EAI were analyzed using the Pearson correlation coefficient (r) and the corresponding probability value (p). Multiple linear regression analysis was performed to examine whether the cytokine/chemokine transcript levels were independently associated with CAI and EAI. To control a possible bias by more than one biopsy of a person the generalized estimating equation (GEE) method was used with an independent and an exchangeable working correlation matrix. Two sided probability values were always computed and an effect was considered statistically significant at a value of p < 0.05.

## Results

### Expression of cytokine/chemokine transcripts in mucosal biopsies of UC patients

In 49 biopsies from 27 UC patients the expression of CXCL8, CXCL10, calgranulin B and CXCL2 transcripts were measured. The median number of transcript copies in all biopsies was 80 (range, 0–600) for CXCL8, 75 (range, 3–339) for CXCL10, 799 (range, 16–10517) for calgranulin B and 353 (range, 10–2484) for CXCL2.

### Correlation of cytokine/chemokine transcript levels with CAI and EAI

**Figure 1:**
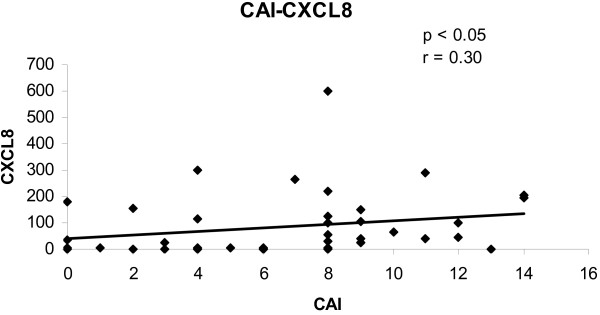
**Linear regression analysis of CXCL8 transcript levels and CAI in all biopsies**. On the x axis, the values of the CAI are given. On the y axis, the cytokine transcript levels are shown. Each single biopsy is characterized by 1 point within the graph. For the graph the related Pearson correlation coefficient (r) and the corresponding probability value (p) are given.

**Figure 2:**
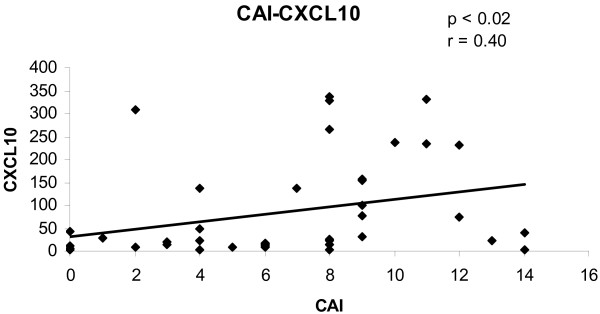
**Linear regression analysis of CXCL10 transcript levels and CAI in all biopsies**. On the x axis, the values of the CAI are given. On the y axis, the cytokine transcript levels are shown. Each single biopsy is characterized by 1 point within the graph. For the graph the related Pearson correlation coefficient (r) and the corresponding probability value (p) are given.

**Figure 3:**
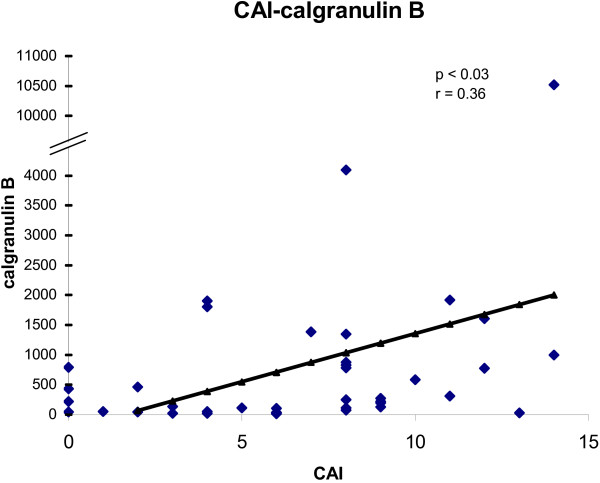
**Linear regression analysis of calgranulin B transcript levels and CAI in all biopsies**. On the x axis, the values of the CAI are given. On the y axis, the cytokine transcript levels are shown. Each single biopsy is characterized by 1 point within the graph. For the graph the related Pearson correlation coefficient (r) and the corresponding probability value (p) are given.

**Figure 4:**
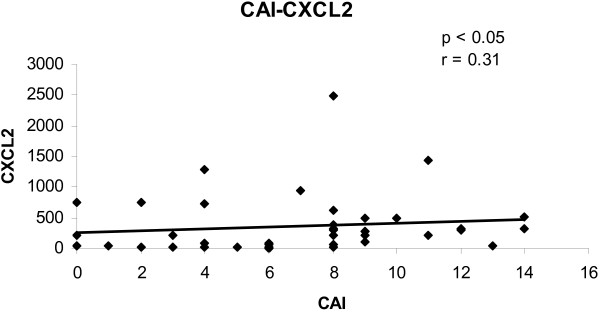
**Linear regression analysis of CXCL2 transcript levels and CAI in all biopsies**. On the x axis, the values of the CAI are given. On the y axis, the cytokine transcript levels are shown. Each single biopsy is characterized by 1 point within the graph. For the graph the related Pearson correlation coefficient (r) and the corresponding probability value (p) are given.

**Figure 5:**
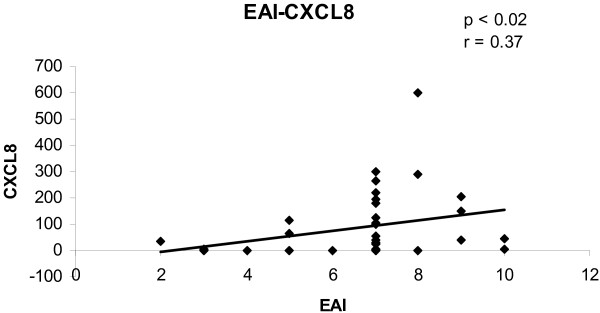
**Linear regression analysis of CXCL8 transcript levels and EAI in all biopsies**. On the x axis, the values of the EAI are given. On the y axis, the cytokine transcript levels are shown. Each single biopsy is characterized by 1 point within the graph. For the graph the related Pearson correlation coefficient (r) and the corresponding probability value (p) are given.

**Figure 6:**
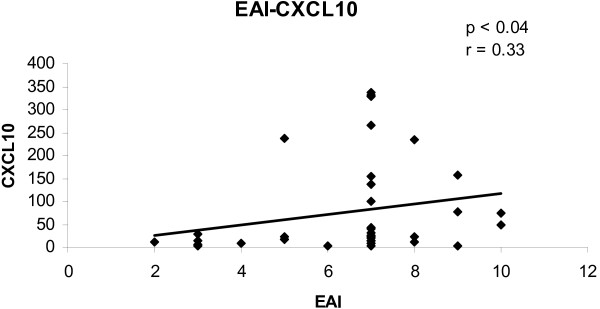
**Linear regression analysis of CXCL10 transcript levels and EAI in all biopsies**. On the x axis, the values of the EAI are given. On the y axis, the cytokine transcript levels are shown. Each single biopsy is characterized by 1 point within the graph. For the graph the related Pearson correlation coefficient (r) and the corresponding probability value (p) are given.

**Figure 7:**
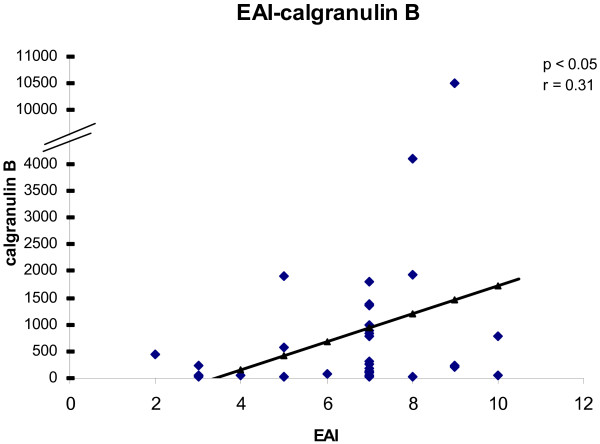
**Linear regression analysis of calgranulin B transcript levels and EAI in all biopsies**. On the x axis, the values of the EAI are given. On the y axis, the cytokine transcript levels are shown. Each single biopsy is characterized by 1 point within the graph. For the graph the related Pearson correlation coefficient (r) and the corresponding probability value (p) are given.

**Figure 8:**
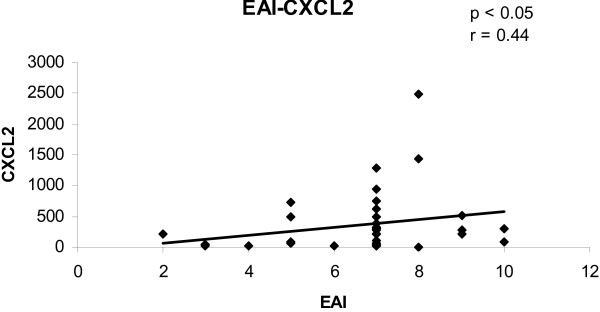
**Linear regression analysis of CXCL2 transcript levels and EAI in all biopsies**. On the x axis, the values of the EAI are given. On the y axis, the cytokine transcript levels are shown. Each single biopsy is characterized by 1 point within the graph. For the graph the related Pearson correlation coefficient (r) and the corresponding probability value (p) are given.

To determine the convergence of cytokine/chemokine transcript levels with the activity of UC, the correlations between cytokine/chemokine transcript levels and clinical and endoscopic disease activity were calculated. The correlations between cytokine/chemokine transcript levels and CAI for all biopsies are shown in figures 1, 2, 3, 4. There was a statistically significant positive correlation between CXCL8 (r = 0.30; p < 0.05), CXCL10 (r = 0.40; p < 0.02), calgranulin B (r = 0.36; p < 0.03) and CXCL2 (r = 0.31; p < 0.05) with CAI. The correlations between cytokine/chemokine transcript levels and EAI for all biopsies are shown in figures 5, 6, 7, 8. Concerning EAI, we could also find significant positive correlations for CXCL8 (r = 0.37; p < 0.02), CXCL10 (r = 0.33; p < 0.04), calgranulin B (r = 0.31; p < 0.05) and CXCL2 (r = 0.44; p < 0.05). Positive correlations indicate that clinical and endoscopic improvement led to a decrease in cytokine levels whereas a deterioration of clinical and endoscopic activity was accompanied by increased cytokine levels.

Correlation analysis was performed to examine a possible internal correlation between CXCL8, CXCL10, calgranulin B and CXCL2. Statistically significant positive correlations were found between CXCL8 and CXCL2 (r = 0.94; p < 0.0001), between CXCL8 and calgranulin B (r = 0.55; p = 0.0002) and between CXCL2 and calgranulin B (r = 0.46; p = 0.002). Linear regression analysis revealed then calgranulin B (p = 0.0008, p = 0.0007) and CXCL10 (p = 0.0412, p = 0.0873) independently correlated with CAI and EAI, respectively.

Regarding the variation of CAI and EAI expressed by CXCL2, CXCL8, CXCL10, and calgranulin B the results of the linear regression for R-square were 0.360 and 0.216, respectively.

## Discussion

Although there has been progress in understanding the pathogenesis of IBD in the last decade, the etiology remains unknown. Therefore, most therapies aim at a symptomatic treatment of inflammation. However, objective assessment of mucosal inflammation and disease activity is often difficult. Neither CAI nor EAI, as established scores, can characterize mucosal inflammation and disease activity to the full extent. The CAI is influenced by patients' perception and may thus reflect clinical symptoms due to other factors than mucosal inflammation and the severity of endoscopic lesions often do not correlate with clinical severity.

The aim of our study was to find a simple and objective means to quantify inflammatory activity in colonic mucosa of UC patients. Therefore, we tried to establish several biomarkers, which can reflect mucosal inflammation in UC.

Thus, we measured the transcript levels of CXCL8, CXCL10, calgranulin B and CXCL2 in biopsies of UC patients and correlated them to CAI and EAI.

The new and substantial finding of our study is the significant positive correlation between transcript levels of CXCL8, CXCL10, calgranulin B and CXCL2 and both CAI and EAI.

Our results agree with the literature. For CXCL8, which is the major attractant and activator of neutrophils [9,10,16], a high expression of mRNA in UC patients with high disease activity has been described [8]. Another group [17], who performed immunohistochemistry and image analysis found enhanced CXCL10 expression in UC. CXCL10 selectively attracts activated T lymphocytes. Calgranulin B is selectively secreted by human monocytes and granulocytes. Using ELISA technique an increased serum level was detected in UC patients with active disease [18]. The same group [19] could also show a greater calgranulin B production in CD in ulcerative and fissural lesions than in uninflamed areas. Concerning CXCL2, which is extremely chemotactic for neutrophils [20], an upregulated expression of mRNA and protein in inflamed gut, predominantly in UC has been shown [1].

These data from the literature strengthen our findings that CXCL8, CXCL10, calgranulin B and CXCL2 are objective indicators of disease activity and severity in UC.

## Conclusion

Therefore, we conclude that measuring transcript levels of CXCL8, CXCL10, calgranulin B and CXCL2 by RT-PCR is a fast, simple and objective method to quantify mucosal inflammation in UC. This approach can give important information in addition to CAI and EAI and thus provide an objective marker of intestinal inflammation. Moreover, using this technique to monitor therapeutic effects of e. g. novel drugs can offer potential improvement in clinical research.

## Competing interests

The authors declare that they have no competing interests.

## Authors' contributions

AZ was involved in the development of methods, performed data and sample collection, data analysis and wrote the paper. TG was involved in the development of methods and carried out the quantitative RT-PCR. MK contributed to the data collection and analysis. AB contributed to the data collection and analysis. UH performed statistical computation. WS contributed substantially to the design of the study and to the interpretation of data. RE developed the original idea of the study, contributed substantially to the design of the study, was involved in data analysis and reviewed the manuscript finally. All authors read and approved the final manuscript.

## Pre-publication history

The pre-publication history for this paper can be accessed here:

http://www.biomedcentral.com/1471-230X/9/13/prepub
